# A vulvar fibroadenoma: A rare presentation of ectopic breast tissue

**DOI:** 10.1111/1346-8138.17683

**Published:** 2025-02-25

**Authors:** Ramiro Alanis Ronquillo, Ixchel Kenia Martínez Velo, Erandy Alicia Salcedo Elguea, Gerardo Torres Barragán, Carmen Itzayana Rodríguez Chaparro

**Affiliations:** ^1^ Internal Medicine Resident ISSSTE General Hospital Juarez City Chihuahua Mexico; ^2^ Division of Basic Sciences, Faculty of Medicine UNAM Mexico City Mexico; ^3^ Obstetrics and Gynecology Department IMSS General Hospital No 6 Juarez City Chihuahua Mexico

**Keywords:** ectopic breast tissue, fibroadenoma, vulva

A 39‐year‐old woman with a 4 × 4 cm vulvar mass of insidious growth, which increased in size over 16‐years in relation to her menstrual cycles. The lesion had a polypoid appearance, regular borders, and firm consistency, and adhered to deep planes, without discoloration or collateral circulation. It was painful to palpation and movement (Figure 1). Histopathological examination after surgical resection reported a benign, biphasic neoplasm with an epithelial component showing ducts and papillae lined by cuboidal and cylindrical cells, without atypia. There was no evidence of necrosis or mitosis, which led to a diagnosis of vulvar fibroadenoma in ectopic mammary tissue (Figure 1).

**FIGURE 1:**
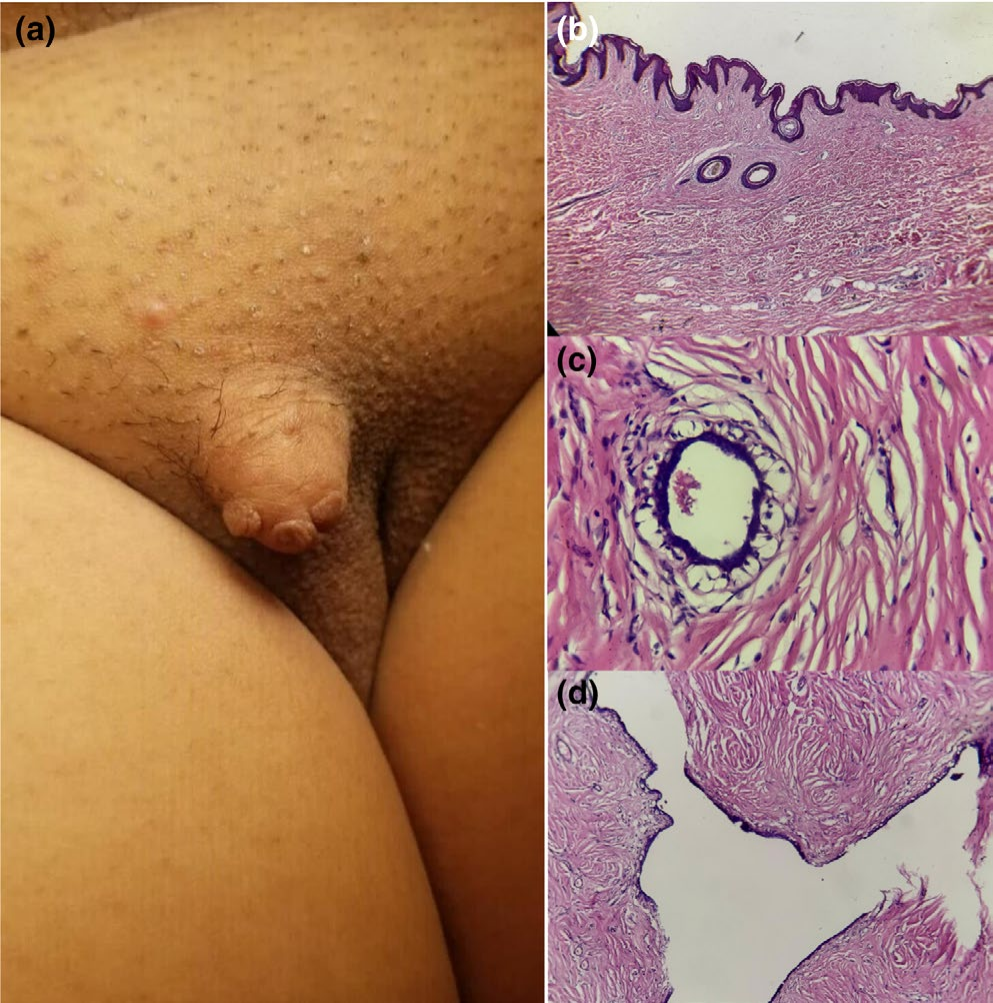
(a) Pedunculated tumor (fibroadenoma) in the upper region of the right labia majora, of hard consistency and well‐defined edges. (b) Epidermis, papillary dermis, reticular dermis, and vulvar adnexal structures. (c) Cuboidal epithelial cells in a monolayer with apocrine metaplasia, surrounded by a monolayer of myoepithelial cells with abundant clear cytoplasm and centrally located nuclei with homogeneous chromatin. (d) Mesenchymal neoplastic proliferation compressing glandular spaces, characteristic of pericanalicular fibroadenoma. b) 4x; c) 40x; d) 10x.

Vulvar fibroadenoma is a rare benign tumor, with approximately 54 cases reported so far. The origin of these tumors has been the subject of debate, with two main theories. The first theory postulates that these tumors arise from ectopic breast tissue due to incomplete regression of the embryonic mammary line; the other theory suggests that they originate in the anogenital glands which are similar to the mammary glands.^1^, ^2^

Clinically, vulvar fibroadenomas typically present as painless, mobile masses in the vulvar region, often confused with cysts or other benign lesions.^2^ Histopathological examination is essential for diagnosis, revealing the typical features of fibroadenoma, including a nodular overgrowth of epithelial and stromal components.^1^, ^3^ Because of the potential for malignant transformation, complete excision with clear margins and close follow‐up is recommended.^3^

Vulvar fibroadenoma is a rare occurrence in dermatology, presenting as a benign neoplasm that is more commonly associated with ectopic breast tissue. Given their rarity, these lesions can be misdiagnosed, especially if they show atypical features such as pseudolactational changes, which can mimic malignancy. It presents with non‐specific symptoms or may be associated with hormonal changes. Diagnosis is supported by microscopic examination, and excision is the ideal treatment.

## CONFLICT OF INTEREST STATEMENT

None declared.

## CONSENT

Informed consent was obtained from the patient for publication of this case report.

## References

[1] Aden D , Saini A , Singh M , Zaheer S . Fibroadenoma vulva: experience based on FNA of vulvar lesion. Cytojournal. 2023;20:12. 37292121 10.25259/Cytojournal_24_2022 PMC10246337

[2] Homsi HA , Sharma A , Przybycin C , Piliang M . Fibroadenoma of the vulva with pseudoangiomatous stromal hyperplasia: a common neoplasm in uncommon site. J Cutan Pathol. 2024;51:583–588. 38695362 10.1111/cup.14613

[3] Noor R , Kumar A , Pervaiz J , Ali R , Sanjna F . Uncommon presentation: a case report on a rare vulvar fibroadenoma. Cureus. 2024;16:e53834. 38465085 10.7759/cureus.53834 PMC10924659

